# Peritoneal tuberculosis mimicking carcinomatous ascites in a child living in a low prevalence country: a case report

**DOI:** 10.1186/s13052-020-0816-6

**Published:** 2020-04-19

**Authors:** Alessandro Rossi, Velia Melone, Rossella Turco, Luigi Camera, Eugenia Bruzzese, Erasmo Miele, Annamaria Staiano, Alfredo Guarino, Andrea Lo Vecchio

**Affiliations:** 1 Department of Translational Medical Sciences, Section of Pediatrics, University of Naples “Federico II”, Via Sergio Pansini, 5, 80131 Naples, Italy; 2 Department of Advanced Biomedical Sciences, Section of Diagnostic Imaging, University of Naples “Federico II”, Naples, Italy

**Keywords:** Ascites, Children, Peritoneal tuberculosis, Ca125, Case report

## Abstract

**Background:**

Ascites can develop as a consequence of a number of diseases in childhood. Despite chronic liver disease is the most common cause, several conditions can lead to ascites also in the absence of liver dysfunction. As non-cirrhotic ascites shows a high degree of overlapping sign and symptoms it is still a challenge for physicians.

**Case presentation:**

A 8-year-old Caucasian girl was referred for fever, vomit and diarrhea occurred over the past few weeks. Physical examination showed timpanitic distension of the abdomen with marked tenderness and increased abdominal wall rigidity. Abdominal imaging showed diffuse ascites and thickened omentum and bowel wall. Blood tests showed increased C- reactive protein levels and decreased lymphocyte count. Specific treatment for inflammatory bowel disease was started. Persisting of ascites required additional investigations. Positive tuberculin skin test and Interferon Gamma release assay (IGRA) as well as increased Ca125 serum concentrations were found. Computed tomography scan showed mediastinal and mesenteric adenopathies and diffuse smooth thickening of the omentum with significant enhancement (omental cake-like). Ascitic fluid analysis revealed high leucocytes and protein levels. Presumptive diagnosis of peritoneal tuberculosis (PTB) was made. Antituberculous treatment resulted in the resolution of ascites and normalization of lymphocyte count and Ca125 serum concentrations.

**Conclusions:**

PTB is still possible in low-prevalence countries. As it is a great mimicker of other abdominal pathology whose treatment might worsen tuberculosis progression, clinical suspicion and adequate screening are required to avoid unnecessary interventions and delayed treatment. Ca125 is a non-specific marker of peritoneal inflammation but it might be helpful in monitoring the treatment response.

## Background

Ascites is defined as the pathologic accumulation of fluid within the peritoneal cavity [1]. Despite data regarding ascites in children have been mostly published in small case series, increased prevalence of hospital admissions for ascites has been reported over the past years in developed countries [2]. The causes of ascites vary according to the age of the patients [3]; they can be classified into two groups: cirrhotic and non-cirrhotic. Cirrhosis from chronic liver disease is the most common cause in infants and children; ascites is the result of (the combination of) portal hypertension, vasodilation and hyperaldosteronism. Clinical presentation include increased abdominal girth and weight, umbilical collateral veins and protuberant and tympanic abdomen. Treatment strategies focus on mobilizing intraperitoneal fluid and correcting the relative systemic hypovolemia (e.g. dietary sodium restriction, diuretics, supplemental albumin) [3, 4]. A number of conditions can lead to non-cirrhotic ascites in childhood. These include: renal disease, heart failure, obstruction (e.g. Budd-Chiari syndrome), infections (e.g. CMV, EBV, tuberculosis), inflammatory bowel disease (IBD), malignancies, omental cyst [5]. Non-cirrhotic ascites can develop as a consequence of secretion of proteinaceous material (e.g. malignancies, infections, IBD), impaired portal blood flow (e.g. Budd-Chiari syndrome, heart failure) or decreased intravascular oncotic pressure (e.g. nephrotic syndrome). Clinical presentation is similar to cirrhotic ascites; additional findings may include fever, vomiting, jaundice, respiratory distress. Besides general treatment (see cirrhotic ascites), specific strategies (e.g. antibiotics, anti-inflammatory drugs, anticoagulants, chemotherapy, surgery) can be considered [3].

Appropriate treatment of ascites is crucial to minimize morbidity from its complications (e.g. compromised ventilation, infections, gastrointestinal hemorrhage, renal failure). Non-cirrhotic ascites constitutes, however, a vast category of causes with a high degree of overlapping sign and symptoms often leading to delayed diagnosis and unnecessary surgery [5].

Currently, Italy is a tuberculosis (TB) low-incidence country. Active TB notified cases account for 4.7% of the pediatric TB cases in Europe [6] with estimated prevalence around 2/100.000 cases in children [7].

Abdominal TB is one of the most common extrapulmonary presentations. It may involve the gastrointestinal tract, peritoneum or the mesenteric lymph nodes. Intestinal TB occurs as a result of ingesting contaminated milk or from swallowing the sputum of active lung disease. Peritoneum involvement has been reported in up to 3.5% of cases of pulmonary TB and comprises 31–58% of cases of abdominal TB; it is usually secondary to haematogenous spread of tubercles from a pulmonary focus [8].

We herein reported on a child with ascites subsequently diagnosed with peritoneal tuberculosis (PTB), highlighting that PTB can mimic other abdominal pathologies causing delayed diagnosis.

## Case presentation

A previously healthy 8-year-old Caucasian girl living in Italy was referred to the general hospital for the occurrence of intermittent fever up to 38 °C (irrespective of antipyretics), vomit and diarrhea (without evidence of blood or mucus) within the previous 10 days. Her mother also referred lack of appetite, weight loss (3 Kg) and abdominal pain dating back to few weeks before. Her medical history revealed no travel outside the country, no definite contact with infected people and an episode of pneumonia 2 years before. No previous abdominal complaints or diarrhea were reported.

On physical examination the girl showed clear sensorium, but appeared restless. Heart rate was 110 per minute, respiratory rate was 26 per minute, blood pressure was 90/50 mmHg, oxygen saturation was 97% (room air), ear temperature was 36 °C. No signs of dehydration were noted. Examination of the cardiovascular and respiratory systems was normal. Abdomen examination revealed timpanitic distension, marked tenderness and increased abdominal wall rigidity. Liver and spleen were not enlarged. Neurologic examination was normal. Height and weight were both at 50° centile per sex and age.

**Fig. 1:**
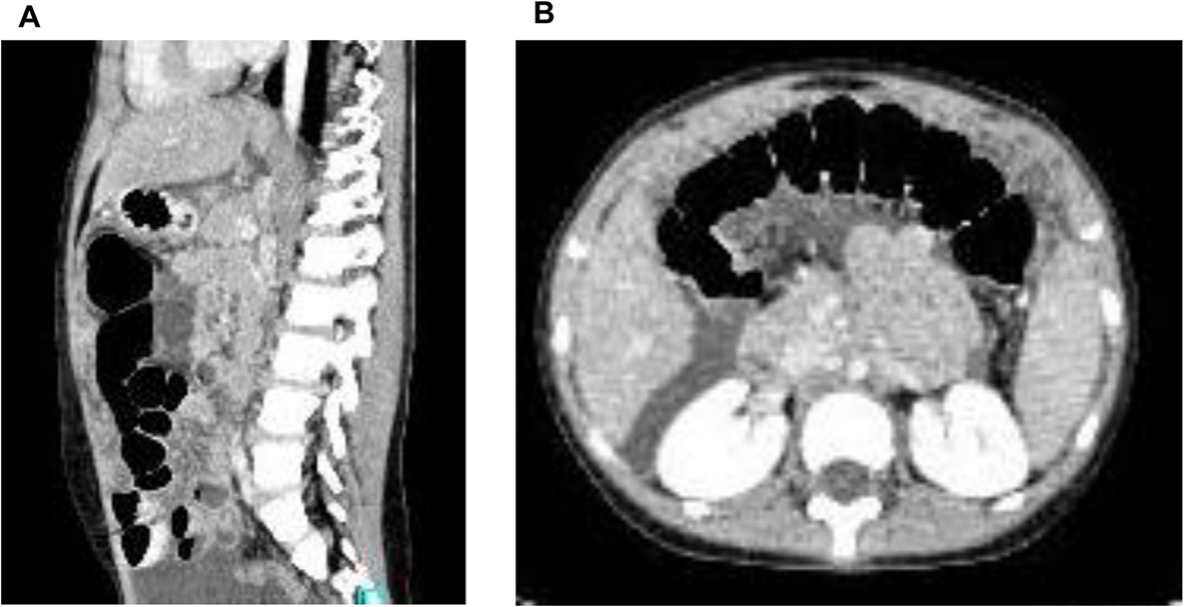
Sagittal (**a**) and axial (**b**) computed tomography of the abdomen and pelvis demonstrating diffuse ascites with simmetrical peritoneum thickening, mesenteric adenopathies and nodularity of the omentum with mild bowel wall thickening and “omental cake-like” appearance

Abdominal radiograph showed bowel loops dilation and some air-fluid levels in the umbilical region with no signs of gastrointestinal perforation. Abdominal ultrasound showed diffuse particulate ascites, thickened and oedematous omentum and thickening of the bowel wall (mean 6.5 mm, Ref <  5 mm). Abdominal computed tomography (CT) scan was also performed showing: diffuse intraperitoneal fluid accumulation (perihepatic, perisplenic, paracolic, Morison’ s pouch, pelvis), mesenteric edema, thickened ileum, colonic and sigma walls with pronounced enhancement (Fig. 1). Chest X-ray revealed accentuated bronchovascular markings. CT of the chest was reported normal.

Patient’s laboratory data are summarized in Table 1. On admission blood tests showed lymphopenia with normal neutrophil count, increased platelet count and C-reactive protein (CRP) levels. On suspicion of possible IBD, specific therapy was started, including methylprednisolone 1 mg/kg/day, metronidazole 30 mg/Kg/day and ceftriaxone 55 mg/Kg/day intravenously. The patient was subsequently referred to the regional reference center. Immediately after treatment her body temperature normalized, CRP levels rapidly decreased (from 132.35 mg/L to 4.69 mg/L within three days) and platelet count normalized within 5–6 days. Lymphocytes count persisted low (800/μL). However, based on clinical and laboratory findings, the diagnosis of IBD was not considered likely. Therefore the treatment was gradually discontinued. Notably, body temperature increased again upon steroid withdrawal.

**Table 1:** Patient’s blood and other specimens tests

BLOOD TESTS		Reference Range	OTHER TESTS		Reference Range
White blood cells (WBC)/μL	6420	5000–15,000	Mantoux TST (mm)	12^a^	< 10.0
Neutrophils/μL	4980	1300–8500	Fecal calprotectin (μg/g)	< 15.0	< 90.0
Lymphocytes/μL	770	1300–8500	Urinalysis	Negative	Negative
Hemoglobin (g/dL)	11.7	10.5–14.0	Blood culture	Negative	Negative
Platelets/μL	601,000	140,000–440,000	Blood smear	Anisocytosis, microcytosis, poikilocytosis, band cells (rare), manteined formula	
C-reactive protein (mg/L)	132.3	0.0–5.0	Ascitic fluid analysis	Leukocites 3979/μL Proteins > 1000 mg/dL SAAG < 1.1 g/dL	
Glucose (mmol/L)	4.5	3.3–6.1	Ascitic fluid citology	Lymphocyte predominance No atipical or bizzare cells	
Iron (μg/dL)	15.0	30–120	ECG	Negative	Negative
Albumin (g/dL)	3.4	3.4–4.8	Cardiac US	Negative	Negative
Total protein (g/dL)	7.0	6.0–8.0	Interferon Gamma Release Assay - IGRA (first evaluation)	0.39	<0.35
Sodium (mEq/L)	138.0	135–145	Interferon Gamma Release Assay - IGRA (second evaluation)	1.62	<0.35
Potassium (mEq/L)	3.5	3.4–5.5	Ziehl-Nielsen (ZN) staining (3 gastric aspirates)	Negative	Negative
Chloride (mEq/L)	97.0	96.0–115.0	Culture for acid-alcohol resistant bacilli (3 gastric aspirates)	Negative	Negative
Calcium (mg/dL)	8.8	8.6–11.0	Polymerase Chain Reaction (PCR) - Gene Xpert (3 gastric aspirates)	Negative	Negative
Creatinine (mg/dL)	0.58	0.30–0.80	Vidal agglutination test	Negative	Negative
Urea (mg/dL)	10.0	10.0–38.0	Wright agglutination test	Negative	Negative
eGFR (ml/min/1.73 m2)	121.38	93–129.6	CMV-IgG (U/mL)	< 5.0	< 12.0
AST (U/L)	24.0	5.0–58.0	CMV-IgM (U/mL)	8.07	< 18.0
ALT (U/L)	10.0	8.0–40.0	HSV I/II-IgG	Positive	Negative
GGT (U/L)	13.0	12.0–64.0	HSV I/II-IgM	Negative	Negative
PT- INR	1.1	0.8–1.2	Rubella-IgG (IU/mL)	85.7	< 9.0
Fibrinogen (mg/dL)	413.0	180.0–400.0	Rubella-IgM (IU/mL)	Negative	Negative
Amylase (U/L)	32.0	10.0–80.0	EBV-VCA-IgG (UA/mL)	137	< 20.0
IgG (mg/dL)	1030.0	650.0–1600.0	EBV-VCA-IgM (UA/mL)	< 10.0	< 20.0
IgA (mg/dL)	286	40.0–350.0			
IgM (mg/dL)	104.0	50.0–300.0			
tTg IgA (CU)	8.4	< 15.0			
BetaHCG (mIU/mL)	< 1.0	< 5.0			
AFP (ng/mL)	0.9	< 15.0			
CEA (ng/mL)	0.6	0.0–4.0			
Ca19–9 (U/mL)	14.7	0.0–37.0			
Ca125 (U/mL)	472	0.0–35.0			
FSH (mU/mL)	0.2	< 5.0			
LH (mU/mL)	< 0.1	< 5.1			
Prolactin (ng/mL)	16.3	3.0–24.0			
Estradiol (pg/mL)	21.0	5.0–20.0			
Testosterone (ng/dL)	< 20.0	< 20.0			

^a^during steroid treatment

Persisting the ascites, extensive work-up was started (Table 1). No signs of hepatopathy, nephropathy or heart disease were noted. A blood culture showed no presence of aerobic or anaerobic bacteria. Blood tests for common infections were negative. Despite still on steroid treatment, Mantoux tuberculin skin test (TST) tested positive (12 mm) at 72 h. TST tested negative in her parents. Interferon Gamma release assay (IGRA) was barely positive (0.39 UI/ml, reference < 0.35 UI/ml) during steroid tapering off and markedly positive (1.62 UI/ml) after steroid withdrawal. Blood tests also showed elevated Ca125 serum levels (472 U/mL).

A new chest X-ray showed accentuated bronchovascular markings with left-sided pleural effusion. A second abdomen ultrasound was also performed, showing diffuse peritoneal effusion, slight wall thickening of the last ileal loop (4 mm, Ref <  5 mm) and hyperechoic mesentery. Second-opinion radiology consultation was requested to reassess the abdominal and chest CT scan, reporting tiny calcified nodules in the Barety’s space, mesenteric lymphadenopathy and diffuse smooth thickening of the omentum with significant enhancement (omental cake-like) (Fig. 1).

**Fig. 2:**
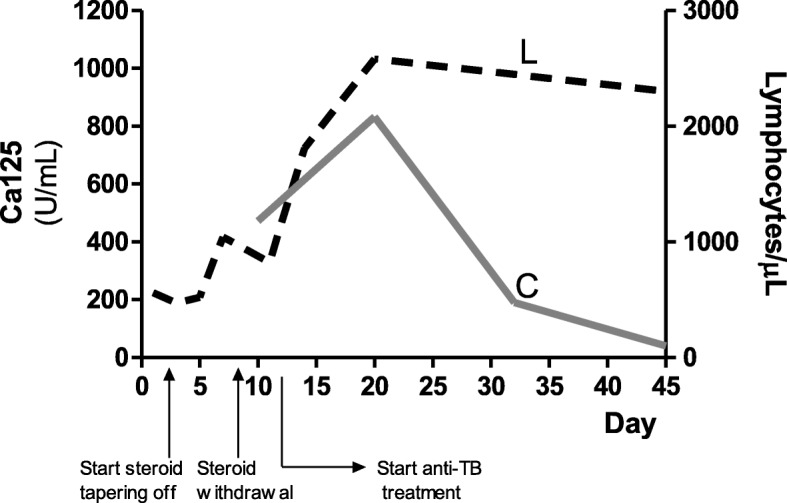
Ca125 (C,) and Lymphocytes (L,) levels before and after the start of anti-TB treatment

Since the underlying etiology remained unclear, gastric aspirates and ascitic fluid were collected for analysis. Three consecutive morning fasting gastric aspirates were collected. Ziehl-Nielsen (ZN) staining, culture for acid-alcohol resistant bacilli (BAAR) and Polymerase Chain Reaction (PCR) for *Mycobacterium tuberculosis (Mtb)* tested negative. Ascitic fluid analysis showed high leucocytes count (3979/ μl) and high protein concentration (> 1000 mg/dl) with low serum-ascites albumin gradient (SAAG, < 1.1 g/dl). Ascitic fluid cytology showed lymphocyte predominance with no atypical or bizarre cells. ZN staining, PCR and culture for *Mtb* tested negative. Although PCR for *Mtb* in ascites was negative, a presumptive diagnosis of peritoneal tuberculosis (PTB) was made, based on omental and intestinal wall thickening, ascites with lymphocyte prevalence, accentuated bronchovascular markings, ileal calcification and pleural effusion in a patient with immunological evidence of TB infection and lack of clinical response to steroid treatment. Due to the young age and the lack of known previous TB exposure, the clinical feature was interpreted as intestinal localization of primary TB infection. Thus, a four-drug regimen was started: rifampin (16 mg/kg/day), isoniazid (12 mg/kg/day), pyrazinamide (32 mg/kg/day), ethambutol (24 mg/kg/day) and continued for 12 months (2 months rifampin, isoniazid, pyrazinamide, ethambutol and 10 months rifampin and isoniazid). The treatment was well tolerated and rapidly effective as the fever and vomit disappeared within 36 h. Within 48 h abdominal complaints resolved and improved general conditions together with increased appetite were noted. Blood tests (including lymphocyte count and Ca125 serum levels) gradually normalized (Fig. 2). Dramatic response to the anti-TB treatment was considered an additional diagnostic criterion. Abdomen Magnetic resonance imaging (MRI) 2 months after the onset of the treatment showed complete disappearance of ascites and all of the lesions.

## Discussion and conclusion

To the best of our knowledge this is the first report of ascites secondary to PTB in a child in Western Europe. Pediatric TB is an emerging health concern in developed countries. According to the World Health Organization (WHO) report, about 1 million children develop TB every year, accounting for 3–6% of the total TB caseload [9]. Extrapulmonary TB occurs in about 15% of cases in Europe [10]. Children show a higher predisposition to the development of extra-pulmonary TB, with more severe clinical course [11, 12]. PTB accounts for 4–10% of all extrapulmonary TB in adults while it is rarely seen in children. It mainly results from lymphohematogenous dissemination spread from a pulmonary focus in developed countries [8]. As many of the signs and symptoms are not specific it is possible that the real incidence of PTB is higher [11]. PTB is reported to be the cause of ascites in only 2% of adult patients; however ascites constitutes the most common presentation of PTB [13]. Despite cirrhotic ascites accounts for the vast majority of ascites in adults, there remain a number of non-cirrhotic causes often leading to delayed diagnosis in children (Table 2). The diagnostic strategy includes physical examination and laboratory evaluation. Abdominal ultrasound is also helpful to evaluate liver and biliary anatomy and the portal system, as well as estimating the volume of the ascitic fluid. However, when the underlying etiology is unclear a diagnostic paracentesis could be necessary [5].

**Table 2:** Main causes of ascites in children

**Chronic liver disease** (cirrhosis)	
**Hepatic non-cirrhotic causes** (e.g. Budd-Chiari syndrome, congenital hepatic fibrosis)	
**Intestinal disorders** (appendicitis, Crohn’s disease, meconium ileus, celiac disease, eosinophilic enterophaty, intestinal atresia, intestinal lymphangectasia, omental cyst, intestinal malrotation)	
**Infections** (CMV, EBV, Tuberculosis)	
**Malignancies** (lymphoma, Wilm’s tumor, germ cells tumors, neuroblastoma, cystic mesothelioma)	
**Urinary disorders** (nephrotic syndrome, obstructive uropathy, bladder rupture, ureterocele)	
**Heart failure**	
**Other** (pancreatitis, inflammatory disorders, metabolic diseases, abdominal trauma, ovarian cyst, ventriculo-peritoneal shunt, thoracic duct trauma)	

The patient herein reported was initially referred for IBD. Despite good response to steroid treatment, some features warranting a wider differential diagnosis were noted: borderline small intestine wall thickness, absence of extraintestinal manifestations, persistent lymphopenia, normal growth pattern, normal fecal calprotectin, persistent ascites. Notably, ascites has only been reported in very few cases of Crohn’s disease, the majority being secondary to associated conditions (e.g. malignancies, portal hypertension) [14, 15]. Therefore other possible causes of ascites were systematically ruled out. Despite negative personal and family history, positive TST and IGRA were found. Differentiating PTB from Crohn’s disease is a major diagnostic challenge in children as immunosuppressants can lead to systemic dissemination of the infection in PTB [16]. The clinical presentation, laboratory, radiological, endoscopic and histopathologic features do not significantly differ between PTB and Crohn’s disease [17]. Interestingly, one recent review showed that the presence of ascites favors PTB while blood in stools and the presence of extraintestinal manifestations favor Crohn’s disease [18].

High Ca125 serum levels were also detected in the present patient. Ca125 is a glycoprotein antigen expressed by tissues of coelemic epithelium including the ovarian epithelium and the mesothelial cells of peritoneum, pleura and pericardium. Its levels are elevated in malignant conditions such as ovarian cancer [19]. However it is a nonspecific marker for malignancy being also elevated in a number of physiologic (e.g. menstruation) and benign inflammatory (e.g. endometriosis, pancreatitis, peritonitis, IBD) conditions [20]. Notably, increased Ca125 serum levels have been reported in both PTB and pulmonary TB and Ca125 can also be used for monitoring anti-TB treatment response [21, 22].

CT findings also show significant overlapping between PTB and ovarian cancer. The typical CT image is the so-called “omental-cake”, that is a diffuse thickening of the omentum to form a mass that can displace the bowel from the abdominal wall [23]. Despite no single CT feature is pathognomonic, there are several findings suggesting PTB: smooth peritoneum with minimal thickening and pronounced enhancement, mesenteric nodules [24].

The gold-standard for the diagnosis of PTB is culturing the *Mtb* from the ascitic fluid or peritoneal biopsies [8]. The SAAG (that is the difference of simultaneously measured concentrations of albumin in the serum and ascitic fluid) is commonly used to differentiate ascites secondary to portal hypertension from alternate etiologies. SAAG > 1.1 g/dl suggests ascites secondary to portal hypertension, whereas a SAAG < 1.1 g/dl is seen in the absence of portal hypertension. However it is not helpful to distinguish between PTB and peritoneal carcinomatosis [25]. Ascitic fluid analysis typically show lymphocitic predominance in PTB and bizarre cells in peritoneal carcinomatosis [26]. ZN staining is positive in only about 3% of cases of PTB. Culture and PCR have higher sensitivity but only in smear-positive patients [27]. Adenosine deaminase (ADA) activity in the ascitic fluid has been proposed as an helpful diagnostic tool for PTB. ADA is an aminohydrolase that converts adenosine to inosine and its activity is more in T than in B lymphocytes; it is increased in tuberculous ascitic fluid because of the stimulation of T cells by the mycobacterial antigens [8]. Unfortunately, this test was not available in the present patient.

Based on the above-mentioned considerations presumptive diagnosis of PTB was made and a four-drug regimen treatment was started in the present patient. Significant clinical and biochemical response to the treatment were considered additional diagnostic criteria for PTB. The subsequent radiological response reinforced the diagnosis [28]. Although PTB is usually secondary to dissemination from a pulmonary focus, primary gastrointestinal TB could not be ruled out in the present case.

One major limitation of the present case was the unavailability of the peritoneal biopsy specimen. The following data were taken into account: positive TST and IGRA, presence of mediastinal and mesenteric adenopathies, abdomen CT findings, ascitic fluid findings. The relative invasiveness of the peritoneal biopsy procedure and the family concerns as well as the need to avoid any further delay to start appropriate treatment were also considered. Notably, failure to isolate *Mtb* from fluid specimens in patients who are suspected of having pulmonary TB based on clinical or radiographic findings does not rule out a diagnosis of TB [29].

PTB is still possible in low-burden countries. Due to the wide differential encompassed by the category non-cirrhotic ascites a systematic approach is required for prompt etiological diagnosis. A high index of suspicion is necessary because PTB is a great mimicker of other abdominal pathology and can mislead physicians to undergo unnecessary interventions and delayed treatment. Ca 125 serum levels are not conclusive for the diagnosis but might be useful in monitoring the treatment response.

## Data Availability

The datasets used and/or analysed during the current study are available from the corresponding author on reasonable request.

## Abbreviations

Ca125: Carbohydrate antigen 125
CT: Computed tomography
IBD: Inflammatory bowel disease
Mtb: *Mycobacterium tuberculosis*
PTB: Peritoneal tuberculosis
IGRA: Interferon Gamma release assay
SAAG: Serum-ascites albumin gradient
TB: Tuberculosis
TST: Mantoux tuberculin skin test
